# Expert perceptions of the relative importance of ecological impacts of biological invasions

**DOI:** 10.1371/journal.pone.0351775

**Published:** 2026-08-19

**Authors:** Laís Carneiro, Gabriel de Oliveira Caetano, Shengyu Wang, Ugo Arbieu, Jane A. Catford, Franck Courchamp

**Affiliations:** 1 Université Paris–Saclay, CNRS, AgroParisTech, Ecologie Société Evolution, Gif-sur-Yvette, France; 2 Department of Geography, King’s College London, London, United Kingdom; 3 Fenner School of Environment & Society, The Australian National University, Canberra, Australia; 4 Chair Biodiversity and Ecosystems, Collège de France, Université PSL, Paris, France; University of Ferrara: Universita degli Studi di Ferrara, ITALY

## Abstract

Invasive species pose a major threat to biodiversity and ecosystem functioning. Although it is widely recognized that invasion impacts differ across habitats and taxonomic groups, a systematic evaluation of their perceived ecological importance has remained unexplored. We surveyed over 400 international experts in invasion science to systematically assess the importance of the 19 different types of ecological impacts across habitats, taxa, and levels of biological organization. We found consistent patterns in expert perceptions, where changes in assemblage structure, population size, and species loss were rated as most important, while others, like soundscape, fire regime, and microclimate, consistently received lower scores. Scores between taxa varied more than scores between habitats, while responses were more consistent among respondents with greater experience in the field. Our results highlight the impact types that would require greater management efforts (i.e., the higher scores), as well as those that could benefit from further research (i.e., the lower scores).

## Introduction

Biological invasions pose a major threat to biodiversity, causing undisputed ecological, economic, and health impacts that disrupt ecosystems and affect human well-being [1,2]. However, the multiplicity of these impacts [3] and their context-dependent nature create challenges in identifying and quantifying invasive species’ effects. For example, the invasion of terrestrial plants [4,5], marine invertebrates [6], freshwater invertebrates [7,8], terrestrial invertebrates [9], and vertebrates [10,11] all demonstrate species-specific and habitat-dependent impact dynamics [12,13]. The visibility and severity of impacts are affected by these context-dependent factors, thereby influencing how they are studied and communicated [14,15].

Although the ecological impacts of invasive species are well documented and many recommendations have been proposed [2], research and management efforts can face challenges in implementing these effectively, especially in setting priorities and quantifying impacts. The urgency of the issue is reflected in Target 6 of the Kunming-Montreal Agreement, which aims to reduce both the introduction and impact of invasive species. Yet limited conservation budgets often force decision-makers to focus only on the most urgent threats [16,17]. Some researchers and practitioners have further noted a frequent mismatch between stated research priorities and the studies that are actually conducted [18]. As a result, key knowledge gaps might persist, limiting our ability to assess and compare the ecological impacts of different invasive species. It remains unclear how to determine which impacts warrant immediate research focus or intervention. This uncertainty is exacerbated by the difficulty in quantifying ecological impacts, which often involve cascading effects across ecological levels, complicating prioritization efforts [19].

In this complex landscape, expert judgment plays a vital role in enabling reliable assessments of invasive species importance [18,20,21]. For example, expert elicitation has helped improve risk assessment [22], uncover key drivers of biological invasions [23], develop monitoring techniques [24], and assess threats to biodiversity [25]. A recent expert elicitation enabled the development of a standardized typology of biological invasion impacts, categorizing 19 impact types across six ecological levels [3]. This provides an opportunity to apply expert elicitation to understand their relative importance, to aid prioritization decisions in research and management.

To assess this, we surveyed 412 international experts in invasion science and asked them to score, from 0 to 10, the importance of each of the 19 types of ecological impacts caused by invasions, while accounting for various ecological contexts. The survey respondents had expertise in different taxonomic groups and habitats. We hypothesised that the 19 types of ecological impacts would not be scored equally, with some receiving consistently higher scores than others. We also expected that different taxonomic groups and ecosystems would receive different scores reflecting that invasive species vary in their traits and per capita effects and that different ecosystems are invaded by different species and are susceptible to different types of impact. Finally, we hypothesised that the perceived importance of different ecological impacts was influenced by the ecological level at which they occur, with some habitats or taxa receiving higher scores at specific levels, such as populations, assemblages, or ecosystems.

## Methods

### Survey design and implementation

The survey was distributed to the international invasion science community via global invasive species mailing lists (IPBES-IAS, ISSG-IUCN, ALIENS-LIST) and the authors’ professional networks. These lists are primarily used by researchers but also include managers and policy makers; however, we did not collect information about respondents’ occupations or other demographic details, and the sample should therefore be interpreted as reflecting the perspectives of a predominantly research-oriented community. Because subscriber numbers for these lists were not available, we could not calculate a response rate. All responses were collected anonymously and assigned a unique identifier for analytical purposes. No personal identifiable information or sensitive data were collected, and participation was entirely voluntary. In accordance with institutional and national guidelines at Université Paris-Saclay, studies based on anonymous, non-interventional surveys that do not collect personal or sensitive data are exempt from formal ethics committee approval. Informed consent was obtained from all participants prior to participation via an introductory statement explaining the purpose of the study and the voluntary nature of participation. The survey was implemented using the Qualtrics online platform and was accessible in six languages: Chinese, English, French, Italian, Portuguese, and Spanish. This multilingual approach was intended to maximize global participation and inclusivity [26,27].

Background questions were included to characterize participant expertise: (i) experience: refers to the years of experience each participant had in the field of biological invasions and participants were grouped into one of five categories (i.e., no experience, < 2 years, 2–4 years, 5–10 years, and >10 years); (ii) habitat of research expertise: terrestrial, marine, freshwater, or any environment; (iii) taxonomic groups of expertise: plants, vertebrates, invertebrates, fungi, microorganisms, or ‘any group’. Respondents could note expertise in more than one habitat type and taxonomic group. This segmentation allowed us to investigate if participants’ backgrounds could shape their perceptions of impact types. Years of experience may reflect both accumulated knowledge and generational differences in attitudes, while habitat and taxonomic expertise capture variation in how impacts are perceived across ecological contexts (e.g., a marine biologist may view fire regime impacts differently to a forest ecologist).

Participants were presented with the 19 ecological impact types described in [3]. They were asked to score (i.e., give the importance of value) each impact type on a scale from 0 (lower importance) to 10 (higher importance), accounting for their main habitat and taxonomic expertise (S1 Appendix). In the context of this survey, importance was defined operationally as the respondent’s perception of the relative ecological significance of a given impact type within their stated area of expertise (i.e., the extent to which the impact matters for populations, assemblages, or ecosystems). As perceptions reflect how individuals observe, understand, and value a specific object, the interpretations of “importance” are inherently value-laden and may vary among individuals (e.g., relating to biodiversity conservation, ecosystem functioning, or other ecological dimensions). Respondents could choose not to score any impact type they did not feel qualified to assess. These responses were excluded from the analysis. To reduce potential bias stemming from the order in which questions were asked, the presentation of impact types was randomized for each respondent (each respondent saw the impacts in a different sequence).

### Statistical analysis

Considering differences in respondent experience and habitats and taxonomic expertise, there were 72 possible score combinations for each of the 19 impact types. Here, we present a general overview for scores across habitats and taxa (S2 Appendix). We analyzed the impact scores to identify their statistical differences across habitats and taxonomic groups using non-parametric analysis and mixed-effects models. We also assessed the difference in scores relative to respondent expertise and the six ecological levels of the impact (i.e., individual, population, species, assemblages, ecosystem functions, and abiotic environment) [3]. We first tested whether the assumptions to use parametric tests were met, to compare impact scores across different categories. We used the Shapiro-Wilk test (shapiro.test function in R) to assess normality, and Levene’s test (leveneTest from the car package) to assess homogeneity of variances. Since the data violated the assumptions of ANOVA, we applied the Kruskal-Wallis test (kruskal.test in R). When the Kruskal-Wallis test indicated significant differences, we performed post hoc pairwise comparisons using the Dunn test (dunn.test package) to determine which specific habitats, taxa, and ecological levels differed significantly in their impact scores.

Because each respondent provided multiple scores, the data have a hierarchical structure with responses nested within individuals. To account for intra-respondent correlation, we fitted linear mixed-effects models including respondent identity as a random intercept. Mixed-effects models were fitted with impact score as the response variable and impact type, respondent experience, habitat, and taxonomic group as fixed effects, with respondent identity included as a random intercept. Statistical significance of fixed effects was assessed using Type III tests, and post hoc pairwise comparisons were conducted using Tukey adjustment.

We conducted a selective inference analysis [28] with LASSO (Least Absolute Shrinkage and Selection Operator) to investigate the association between impact scores and each associated impact type, respondent experience, impacted habitat, and invasive taxon (selectiveInference package). The LASSO model was fitted using least angle regression with dummy variables created for each value in each category (impact type, experience, habitat, and taxa) as predictors. LASSO is a regression technique that shrinks model coefficients while fitting the model, effectively reducing the coefficients of non-important variables to zero [28]. Because of the relatively large number of predictors and their categorical structure, LASSO was used as an exploratory variable selection approach to identify dominant patterns in the data. However, as this method does not account for the hierarchical structure of the data, results are interpreted cautiously and not used for primary inferential conclusions. All statistical analyses were conducted in R (version 4.3.3).

## Results

**Fig 1:**
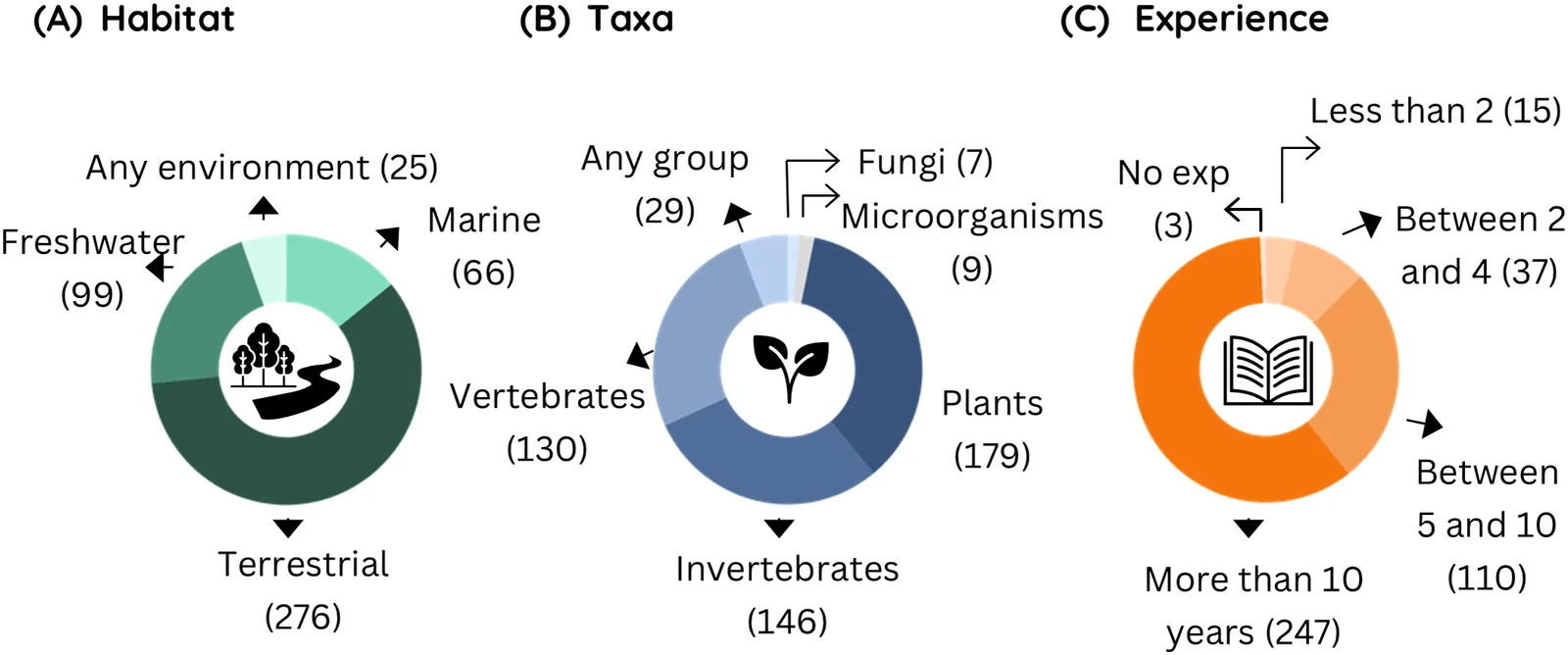
Number of respondents per category. (A) Experience, (B) habitat, and (C) invasive taxa. The numbers in parentheses represent the number of respondents with expertise in each group within each category.

We received responses from 412 respondents between May 2024 and September 2024. Most respondents had expertise in terrestrial ecosystems (n = 276), followed by freshwater ecosystems (n = 99) then marine ecosystems (n = 66) (Fig 1). For taxonomic expertise, the number of respondents was relatively balanced across major groups (plants, n = 179; invertebrates, n = 146; vertebrates, n = 130, whereas representation was lower for fungi (n = 7) and microorganisms (n = 9). The majority of respondents had more than five years of experience in the field of invasion sciences (n = 357).

**Fig 2:**
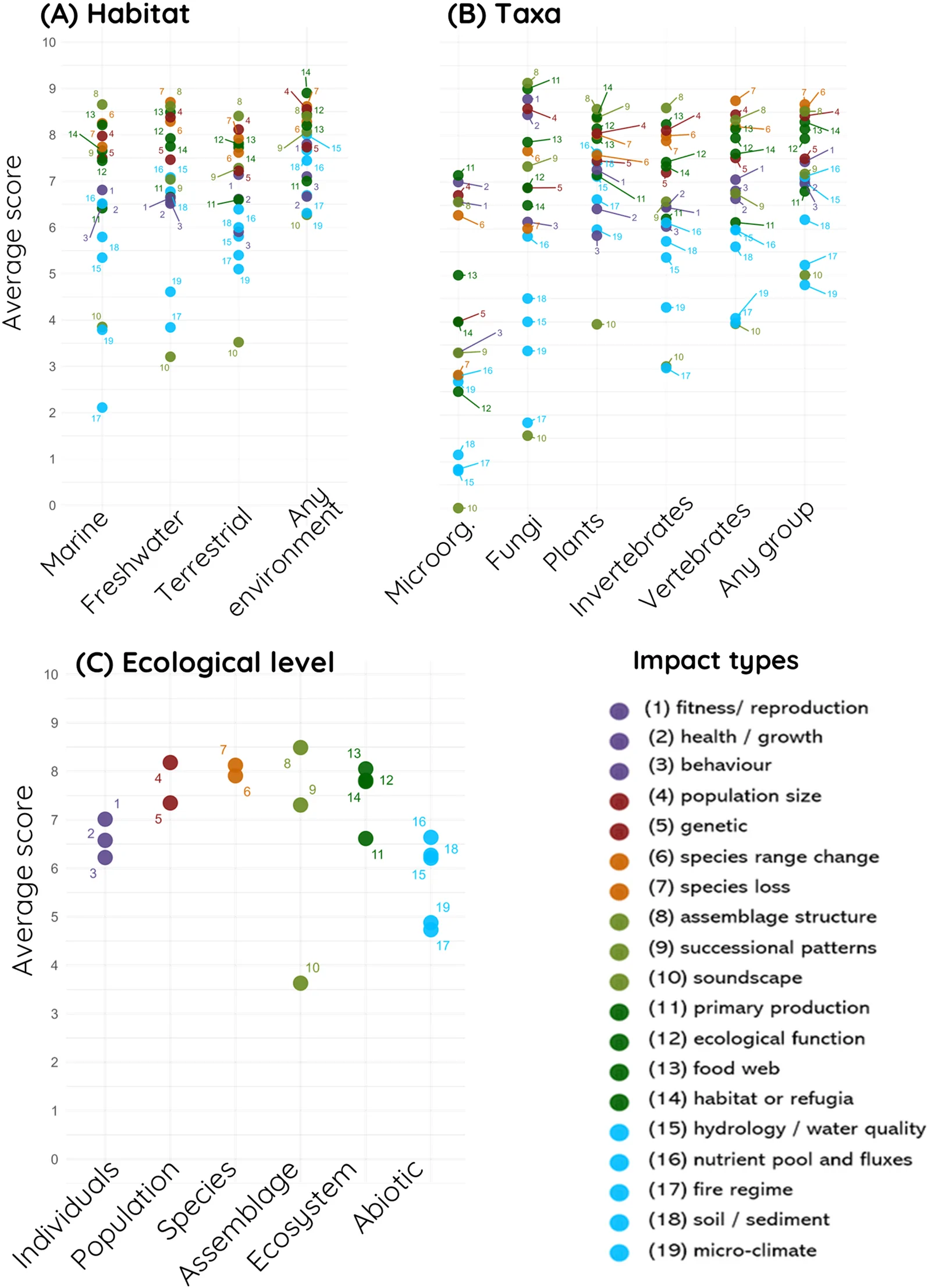
Average impact scores of invasive species. (A) habitat, (B) taxonomic group, and (C) ecological levels, represented by colored dots. The colors denote the impact types across the ecological levels. The numbers refer to the corresponding impact types (in parentheses) in the legend.

On average, the scores for all impact types were higher than the possible neutral average of 5.0, with an overall mean of 6.92 (±3.05), indicating that experts view the impacts of biological invasions as important (S1 Fig). Overall, the top-ranked impact types, regardless of habitat, taxa, or experience, were assemblage structure, population size, and species loss, while soundscape, fire regime, and microclimate were rated the lowest (Fig 2).

**Fig 3:**
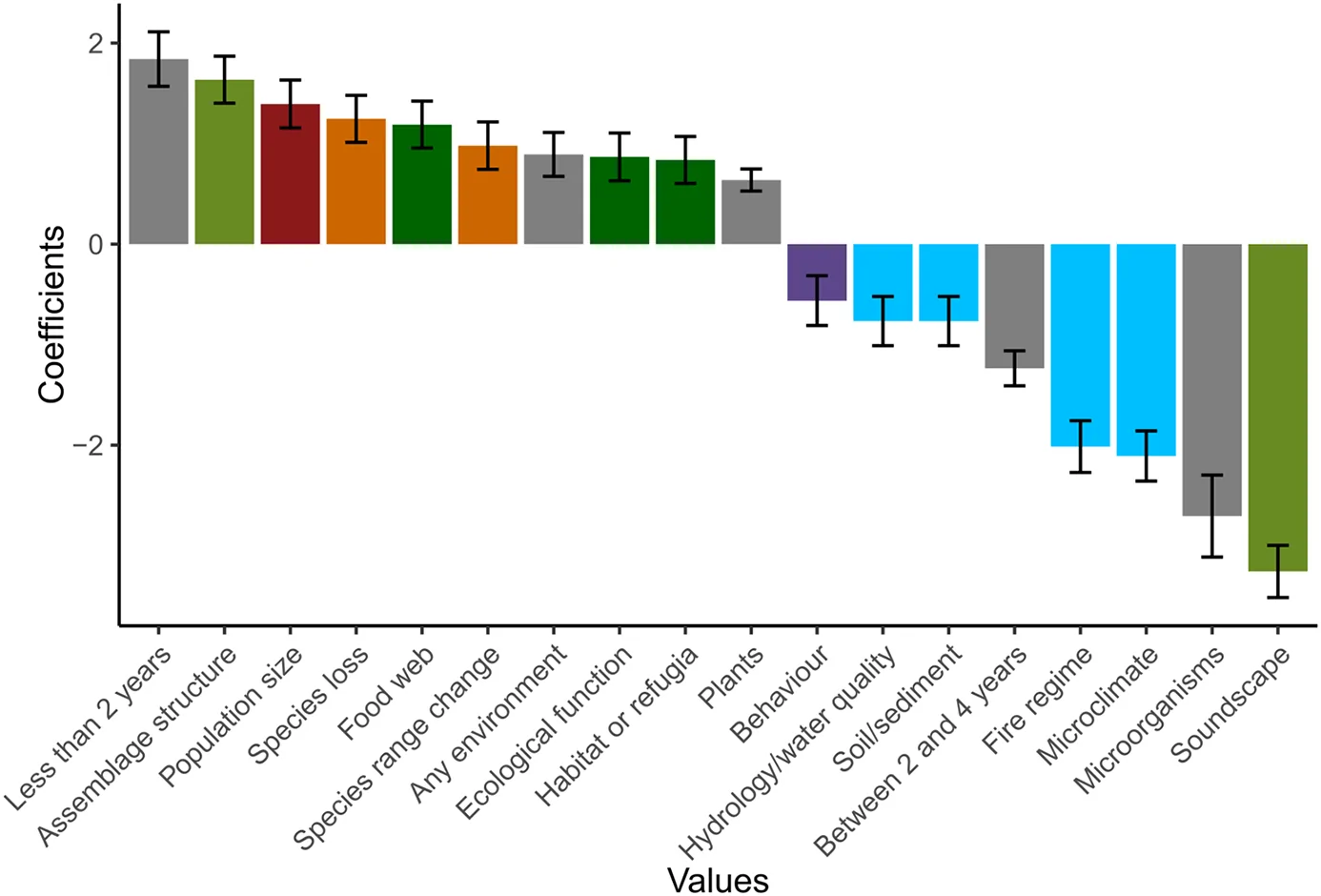
Coefficients of a selection-aware LASSO regression between impact scores and impact type, respondent experience, impacted habitat, and invasive taxonomic group. 95% coefficient intervals are displayed by the whiskers on each bar. Each coefficient represents how much the value for a parameter increases or decreases the impact scores on average, compared to other values in the same parameter. For example, the variable Less than 2 years has a coefficient of 1.8, which means researchers with less than 2 years of experience tend to score impacts 1.8 points higher than researchers with other levels of experience. Values not included in the graph had a coefficient of zero. Colors represent ecological levels of impacts, see Fig 2. Responded experience, impacted habitat and invasive taxonomic group categories are represented in grey.

The LASSO model had an R^2^ of 0.16, indicating that most of the variation in impact scores was due to unmeasured factors. Exploratory LASSO results showed patterns broadly consistent with the dominant influence of impact type (Fig 3 and S2 and S3 Appendix). Accounting for repeated ratings by the same respondent, mixed-effects models indicated substantial within-respondent clustering of scores. After accounting for this structure, impact type remained a strong predictor of perceived importance (S2 and S3 Appendix).

Among habitats, any environment received slightly higher score values (Fig 2). The order of importance for impact types was also variable, particularly between marine and terrestrial ecosystems (S2 and S3 Appendix). For instance, assemblage structure was consistently ranked highest across terrestrial and marine habitats, while species loss had the highest importance in freshwater habitats (S1 Fig, S2 and S3 Appendix). Microorganisms tended to receive lower scores than for other taxonomic groups (Fig 2), although these differences were not supported after accounting for within-respondent clustering (S2 and S3 Appendix). Impact scores differed among ecological levels, with abiotic impacts generally receiving lower scores than other levels, while species-level impacts were among the highest (Fig 2, S2 and S3 Appendix). Some contrasts among ecological levels remained evident after accounting for clustering, particularly between abiotic and biotic levels.

Impact scores also showed limited variation across experience levels (S2 and S3 Appendix). Less experienced respondents tended to assign slightly higher scores (Fig 2 and 3), but these differences were not supported after accounting for within-respondent clustering (S2 and S3 Appendix).

## Discussion

Our study, based on a survey of  > 400 international experts in invasion science across various career stages and expertise spanning all habitats and taxonomic groups, assessed the relative importance of the 19 ecological impact types of invasive species and revealed strong context-dependency. While it is well-established that impacts from invasive species can differ across systems – such as vertebrates in terrestrial ecosystems versus plants in freshwater – our results provide a much-needed demonstration of such variation, whilst also highlighting patterns that are consistent across habitats, taxa, and ecological levels of impacts. Indeed, certain impact types, such as changes in assemblage structure and population size, were consistently given higher importance. Other impacts, like on soundscape and microclimate, consistently received lower scores. Considering that most evidence on the impacts of biological invasions comes from studies at the population level [29], it is not surprising that changes in population size were ranked among the highest impact types. Species loss and changes in assemblage structure are also closely linked to population size changes, because one can lead to the other, and sometimes they can be more visible and easier to detect.

Previous reviews and syntheses also found that changes in species abundance were the most frequently reported impacts, followed by effects on community composition and diversity [30–32]. This mirrors the pattern observed for the ecological levels, where population, species, and assemblages had overall higher scores than individuals, ecosystem functions, and abiotic environment, suggesting that expert importance attributions align with the most studied impacts. This alignment between expert-assigned importance scores and research effort likely reflects a targeted allocation of scientific resources rather than a circular cognitive bias. In a mission-driven discipline, it is both expected and reassuring that the scientific community naturally directs the highest volume of research toward the impacts globally perceived as the most important.

Some impact types were perceived by experts as less important, possibly reflecting both a lower current ecological relevance and the limited evidence currently available to assess them. For example, changes in fire regimes or microclimatic conditions can be difficult to detect, particularly when invasives replace native species that possess similar functional traits [33,34]. Likewise, impacts on the soundscape are still poorly documented, especially in non-vertebrate groups [35], and the evidence that exists for vertebrates is mostly indirect, through behavior [36]. These low scores of impact types also might reflect a lack of convergent evidence, rather than an absence of effects, which lowers their importance compared to other impact types.

Given the inherent ecological differences among terrestrial, freshwater, and marine ecosystems, variation in impact scores across these environments was expected. No single habitat consistently received higher scores across all impact types, suggesting that the different importance attributions for the impacts depend on the ecological context. However, after accounting for within-respondent clustering, differences among habitats were not strongly supported, indicating that these patterns should be interpreted cautiously. For certain impacts, such as changes to fire regimes, these differences remain ecologically intuitive, as this impact is more relevant in terrestrial systems than in marine environments.

Microorganisms consistently received lower impact scores across all categories. This may reflect the inherent resilience of microbial communities, which often show high functional redundancy, strong environmental filtering that limits successful invasions, and rapid turnover rates that help buffer ecological disruptions [37,38]. This trend may also partially reflect the limited representation of microbial experts in our sample, and/or the broader knowledge gaps in the literature [2,39], although this was not apparent for invasive fungi, experts of which were also underrepresented in our survey. Nevertheless, these differences were not strongly supported after accounting for respondent-level variation. Plants, on the other hand, tended to receive slightly higher impact scores than all other taxa. This could reflect their foundational role in ecosystems as primary producers and structural elements, influencing food webs and habitat configuration across a broad range of systems.

We found limited evidence that expert experience strongly influenced impact scores after accounting for within-respondent clustering. Although less experienced respondents tended to assign slightly higher scores in descriptive patterns, these differences were weak, suggesting that perceptions of impact importance are broadly consistent across experience levels. This nonetheless highlights the value of recording respondent experience level in expert-based assessments.

While our study offers a useful synthesis of expert knowledge, it also reflects current research patterns and evidence availability. Importantly, the scores reflect the perceived ecological importance of impact types in each context and allow for comparisons between impacts, but they do not reflect their magnitude of impacts, which is outside the scope of this study. Crucially, these scores must be interpreted as an intrinsic valuation of impact importance (e.g., inherently prioritizing fire regime alterations over soundscape changes), rather than a measure of the severity of each of any given impact, keeping in mind the context-dependent nature of invasion impacts [40]. Participants could choose not to score certain impacts, meaning that those who did were likely more knowledgeable about them. Given the low variability explained by the LASSO model, it is likely that experts had different examples in mind when evaluating the same type of impact. This is because impacts within a category can differ in key attributes such as detectability, immediacy, and severity, leading to varied assessments. To better understand this variability, future surveys should include variables capturing the breadth of respondents’ expertise, such as demographic background, cultural values, research experience, and academic training.

Because our survey did not provide a standardized definition of “importance” and collected limited respondent information, the resulting scores should be interpreted as collective perceptions within a science-oriented community. The intrinsic importance of an ecological impact lacks purely physical measure; it is inherently normative. Consequently, even though one can attribute an average perceived importance in the field, individual perception can vary based on an expert’s experience, their focus on fundamental versus applied management research, and underlying personal biases. However, reliance on value-based expert elicitation should be viewed as a methodological necessity rather than a limitation. This is particularly true in mission-driven fields like conservation biology, which are inherently value-laden [41–43]. Therefore, we interpret the scores as reflecting the collective expertise of over 400 specialists, rather than being primarily driven by individual biases. Of note, the survey design precluded us from calculating a formal response rate or a precise breakdown of researcher versus management profiles, though our outreach naturally leaned toward academic circles. Finally, there is a need to balance research and management priorities: while management actions may focus on highly ranked impacts (e.g., preventing species loss), research should be encouraged to address lower-ranked but potentially underestimated impacts (e.g., fire regime, microclimate). These insights can guide subsequent quantification efforts and inform invasive species management and policy. Ultimately, these scores represent a contemporary snapshot of the agreement tendency within the broader invasion science community. Because perceived impact importance will evolve alongside accumulating empirical knowledge, repeating this survey longitudinally could provide a valuable mechanism to track changes in perceptions and scientific priorities over time.

## Conclusion

This study presents a scoring system to assess the perceived relative importance of ecological impacts caused by biological invasions, grounded in a unified and standardized typology of impact types, and reflecting the current knowledge- and expertise-based value of each impact from experts. We found that some impact types are consistently considered more important across habitats and taxa, based on consistent patterns across respondents. This system reflects current research patterns, highlights the impact types most often considered important, and provides a basis for tracking how these assessments change over time. As control measures against biological invasions become more prevalent, the focus of actions may shift, altering the perceived importance of impacts. This expert elicitation approach provides a practical and flexible tool to help prioritize research and inform potential future shifts in management priorities and ecological impact assessments in the context of global conservation targets. As the field evolves and new data emerge, our framework can be refined and expanded to support adaptive, evidence-based strategies in biodiversity conservation.

## Supporting information

S1 Fig Expert scores for all impact types. Box plots for each impact type according to the (A) invaded habitat and (B) invasive taxa. The black solid line is the overall average of all scores, the red dots are the averages of each impact type, and the dotted lines are their standard deviation. Each transparent dot represents an impact assessment made by one expert. The colors refer to the six different ecological levels of impact types, i.e., individual (purple), population (red), species (orange), assemblages (light green), ecosystem functions (green), and abiotic environment (blue).
(TIF)

S1 Appendix Ecological impacts of biological invasions survey. (PDF)

S2 Appendix Non-parametric tests and exploratory LASSO models. (PDF)

S3 Appendix Mixed-effects model analysis. (PDF)

S1 File Dataset. (XLSX)
