# Supplementary material for: Expert perceptions of the relative importance of ecological impacts of biological invasions
Source: PLoS One. 2026 Aug 19;21(8):e0351775. doi: 10.1371/journal.pone.0351775 (PMC13489405; doi:10.1371/journal.pone.0351775)
Supplement: Supplementary file 1 [file pone.0351775.s002.pdf]

Supporting Information

Expert perceptions of the relative importance of ecological impacts of biological invasions

S1 Appendix. Ecological impacts of biological invasions survey.

Ecological impacts of biological invasions

We are developing a framework to weigh the importance of the different types of impacts associated with biological invasions. The types of impacts were already assessed by more than 60 scientists in a two-step Delphi process, which allowed us to produce this survey, directed to the entire invasion biology community. This survey has five questions and should take only a few minutes to complete. Your participation is important. Thank you for contributing!

Before participating in this study, please be aware of the following: Your participation is voluntary. You are free to stop at any time, without any justification. Your consent does not release those responsible for this study from their responsibilities. You retain all of your rights guaranteed by law. Your identity will remain strictly confidential.

Please confirm whether you agree to participate:

- I freely and voluntarily agree to participate in this survey
- I do not agree

1- How long have you been working in the field of biological invasions?

- Less than 2 years
- Between 2 and 4 years
- Between 5 and 10 years
- More than 10 years
- No experience

2- What is your main ecosystem of research?

- Marine
- Freshwater
- Terrestrial
- Any environment

3- What is the main target species of your research?

- Microorganisms

- Fungi
- Plants
- Invertebrates
- Vertebrates
- Any group

4- Please provide an assessment of the importance (0 low importance, 10 high importance) of each of the impact types caused by biological invasions listed below, based on your main ecosystem and target species expertise:

**Impact type**	0	1	2	3	4	5	6	7	8	9	10	Don't know
fitness and/or reproduction												
health and/or growth												
behavioural												
population size												
genetic diversity												
species range												
species loss												
assemblage structure												
successional patterns												
soundscape												
primary production												
ecological function												
food web												
habitat or refugia												
hydrology / water quality / soil moisture												
nutrient pool and fluxes												
fire regime												
soil / sediment												
micro-climate												

5- The results will be anonymous, but you can still add your email address here if you want to receive the results. You can also provide us with comments on your answers above if you wish.
