# Supplementary material for: Expert perceptions of the relative importance of ecological impacts of biological invasions
Source: PLoS One. 2026 Aug 19;21(8):e0351775. doi: 10.1371/journal.pone.0351775 (PMC13489405; doi:10.1371/journal.pone.0351775)
Supplement: Supplementary file 2 [file pone.0351775.s003.pdf]

Supporting Information

Expert perceptions of the relative importance of ecological impacts of biological invasions

S2 Appendix. Non-parametric tests and exploratory LASSO models.

Table S1: Coefficients of a selection-aware LASSO regression between impact scores and significant values of impact type, research experience, impacted habitat, and invasive taxa. LCI = Lower Confidence Interval, UCI = Upper Confidence Interval.

Parameter	Value	Coefficient	LCI	UCI	*p*-value
Experience	Less than 2 years	1.841	1.569	2.112	<0.001
Impact Type	Assemblage structure	1.635	1.402	1.869	<0.001
Impact Type	Population size	1.393	1.156	1.632	<0.001
Impact Type	Species loss	1.247	1.012	1.480	<0.001
Impact Type	Food web	1.188	0.955	1.422	<0.001
Impact Type	Species range change	0.979	0.744	1.215	<0.001
Habitat	Any environment	0.892	0.674	1.110	<0.001

Impact Type	Ecological function	0.868	0.630	1.105	<0.001
Impact Type	Habitat or refugia	0.837	0.604	1.071	<0.001
Taxa	Plants	0.637	0.528	0.747	<0.001
Impact Type	Behaviour	-0.564	-0.810	-0.314	0.0002
Impact Type	Hydrology/water quality	-0.766	-1.010	-0.521	<0.001
Impact Type	Soil/sediment	-0.767	-1.010	-0.521	<0.001
Experience	Between 2 and 4 years	-1.235	-1.409	-1.061	<0.001
Impact Type	Fire regime	-2.014	-2.272	-1.757	<0.001
Impact Type	Micro-climate	-2.107	-2.358	-1.858	<0.001
Taxa	Microorganisms	-2.705	-3.113	-2.298	<0.001
Impact Type	Soundscape	-3.256	-3.516	-2.997	<0.001

Table S2: Results from Dunn-test to assess the differences between habitat types. Values correspond to the Z statistics and the adjusted p-value in parentheses.

**Habitat type**	Freshwater	Marine	Terrestrial

Any environment	3.21 (0.004)*	4.88 (0.00)*	4.95 (0.00)*
Freshwater		2.76 (0.02)*	2.77 (0.02)*
Marine			-0.86 (1.00)

Table S3: Results from Dunn-test to assess the differences between groups. Values correspond to the Z statistics and the adjusted *p*-value in parentheses.

**Groups**	Fungi	Invertebrates	Microorganisms	Plants	Vertebrates
Any group	1.85 (0.48)	4.51 (0.00)*	9.67 (0.00)*	-0.31 (1.00)	2.89 (0.03)
Fungi		0.48 (1.00)	6.47 (0.00)*	-2.22 (0.20)	-0.41 (1.00)
Invertebrates			8.18 (0.00)*	-9.03 (0.00)*	-2.79 (0.04)
Microorganisms				-10.74 (0.00)*	-9.00 (0.00)*
Plants					5.91 (0.00)*

Table S4: Results from Dunn-test to assess the differences between ecological levels. Values correspond to the Z statistic and the adjusted *p*-value in parenthesis.

**Ecological levels**	Individual	Population	Species	Assemblage	Ecosystem
Population	-11.16 (0.00)*				
Species	-14.64 (0.00)*	-3.06 (0.02)*			
Assemblage	4.04 (0.00)*	-7.42 (0.00)*	-10.83 (0.00)*		
Ecosystem	-11.09 (0.00)*	2.00 (0.3433)	5.59(0.00)*	-6.62 (0.00)*	

Abiotic	-5.52 (0.00)*	-17.06 (0.00)*	-20.96 (0.00)*	-9.94(0.00)*	-18.67 (0.00)*

Table S5: Results from Dunn-test to assess the differences between experiences. Values correspond to the Z statistics and the adjusted *p*-value in parenthesis.

**Experience**	No experience	Less than 2 years	Between 2 and 4 years	Between 5 and 10 years
Less than 2 years	3.61 (0.00)*			
Between 2 and 4 years	-2.86 (0.02)*	-14.87 (0.00)*		
Between 5 and 10 years	-0.21 (1.00)	-10.05 (0.00)*	-8.85 (0.00)*	
More than 10 years	-0.08 (1.00)	10.20 (0.00)*	-10.02 (0.00)*	-0.62 (0.80)
