# Supplementary material for: Expert perceptions of the relative importance of ecological impacts of biological invasions
Source: PLoS One. 2026 Aug 19;21(8):e0351775. doi: 10.1371/journal.pone.0351775 (PMC13489405; doi:10.1371/journal.pone.0351775)
Supplement: Supplementary file 3 [file pone.0351775.s004.pdf]

# Supporting Information

# Expert perceptions of the relative importance of ecological impacts of biological invasions

## S3 Appendix. Mixed-effects model analysis.

**Table S1.** Mixed-model specification and clustering summary

| Item | Value |
|---|---|
| Response | Score (0-10) |
| Fixed effects | Impact type, Environment, Group, Experience |
| Random effect | Respondent ID (random intercept) |
| N observations | 9,529 |
| N respondents | 412 |
| ICC (respondent level) | 0.327 |
| Model formula | score ~ impact_type + Environment + Group + Experience + (1 \| ID) |

**Table S2.** Type III tests from linear mixed-effects model

| Term | NumDF | DenDF | F | p |
|---|---|---|---|---|
| Impact type | 18 | 9097.15 | 158.49 | <0.001 |
| Environment | 3 | 4016.08 | 0.79 | 0.498 |
| Group | 5 | 4338.97 | 2.08 | 0.065 |
| Experience | 4 | 391.38 | 2.36 | 0.053 |

**Table S3.** Tukey-adjusted pairwise contrasts among habitats

| Contrast | Estimate | SE | z | p adj |
|---|---|---|---|---|
| Any environment - Freshwater | 0.321 | 0.261 | 1.23 | 0.606 |
| Any environment - Marine | 0.448 | 0.281 | 1.59 | 0.383 |
| Any environment - Terrestrial | 0.334 | 0.257 | 1.30 | 0.563 |
| Freshwater - Marine | 0.127 | 0.137 | 0.92 | 0.793 |
| Freshwater - Terrestrial | 0.013 | 0.090 | 0.14 | 0.999 |
| Marine - Terrestrial | -0.114 | 0.134 | -0.85 | 0.830 |

**Table S4.** Tukey-adjusted pairwise contrasts among taxonomic groups

| Contrast | Estimate | SE | z | p adj |
|---|---|---|---|---|
| Any group - Fungi | 0.356 | 0.403 | 0.88 | 0.951 |
| Any group - Invertebrates | 0.273 | 0.241 | 1.13 | 0.869 |

| | | | | |
|---|---|---|---|---|
| Any group - Microorganisms | 1.243 | 0.503 | 2.47 | 0.133 |
| Any group - Plants | 0.078 | 0.231 | 0.34 | 0.999 |
| Any group - Vertebrates | 0.205 | 0.240 | 0.86 | 0.957 |
| Fungi - Invertebrates | -0.083 | 0.338 | -0.25 | 1.000 |
| Fungi - Microorganisms | 0.887 | 0.484 | 1.83 | 0.443 |
| Fungi - Plants | -0.278 | 0.337 | -0.83 | 0.963 |
| Fungi - Vertebrates | -0.151 | 0.342 | -0.44 | 0.998 |
| Invertebrates - Microorganisms | 0.970 | 0.456 | 2.13 | 0.273 |
| Invertebrates - Plants | -0.195 | 0.095 | -2.05 | 0.316 |
| Invertebrates - Vertebrates | -0.068 | 0.095 | -0.72 | 0.980 |
| Microorganisms - Plants | -1.166 | 0.452 | -2.58 | 0.103 |
| Microorganisms - Vertebrates | -1.038 | 0.457 | -2.27 | 0.206 |
| Plants - Vertebrates | 0.128 | 0.094 | 1.36 | 0.750 |

**Table S5.** Tukey-adjusted pairwise contrasts among experience levels

| **Contrast** | **Estimate** | **SE** | **z** | **p adj** |
|---|---|---|---|---|
| 2-4 y - 5-10 y | -0.768 | 0.342 | -2.25 | 0.163 |
| 2-4 y - <2 y | -1.424 | 0.548 | -2.60 | 0.071 |
| 2-4 y - >10 y | -0.781 | 0.318 | -2.46 | 0.100 |
| 2-4 y - No exp | -1.378 | 1.076 | -1.28 | 0.704 |
| 5-10 y - <2 y | -0.655 | 0.491 | -1.33 | 0.670 |
| 5-10 y - >10 y | -0.013 | 0.205 | -0.06 | 1.000 |
| 5-10 y - No exp | -0.609 | 1.049 | -0.58 | 0.978 |
| <2 y - >10 y | 0.642 | 0.475 | 1.35 | 0.658 |
| <2 y - No exp | 0.046 | 1.133 | 0.04 | 1.000 |
| >10 y - No exp | -0.596 | 1.041 | -0.57 | 0.979 |

**Table S6.** Tukey-adjusted pairwise contrasts among ecological levels

| **Contrast** | **Estimate** | **SE** | **z** | **p adj** |
|---|---|---|---|---|
| Abiotic - Assemblage | -0.702 | 0.074 | -9.53 | <0.001 |
| Abiotic - Ecosystem | -1.788 | 0.066 | -27.16 | <0.001 |
| Abiotic - Individual | -0.816 | 0.073 | -11.19 | <0.001 |
| Abiotic - Population | -1.955 | 0.082 | -23.88 | <0.001 |
| Abiotic - Species | -2.230 | 0.081 | -27.69 | <0.001 |
| Assemblage - Ecosystem | -1.086 | 0.075 | -14.40 | <0.001 |
| Assemblage - Individual | -0.113 | 0.082 | -1.39 | 0.733 |
| Assemblage - Population | -1.252 | 0.090 | -13.97 | <0.001 |
| Assemblage - Species | -1.528 | 0.088 | -17.27 | <0.001 |
| Ecosystem - Individual | 0.972 | 0.074 | 13.06 | <0.001 |
| Ecosystem - Population | -0.166 | 0.083 | -2.00 | 0.342 |
| Ecosystem - Species | -0.442 | 0.082 | -5.40 | <0.001 |
| Individual - Population | -1.139 | 0.089 | -12.81 | <0.001 |
| Individual - Species | -1.415 | 0.088 | -16.14 | <0.001 |
| Population - Species | -0.276 | 0.095 | -2.90 | 0.043 |

**Table S7.** Fixed effects from main mixed model. Full impact-type coefficients are provided in csv file.

| **Term** | **Estimate** | **SE** | **t** | **p** |
|---|---|---|---|---|
| Intercept | 5.787 | 0.421 | 13.74 | <0.001 |
| Environment: Freshwater | -0.265 | 0.265 | -1.00 | 0.317 |
| Environment: Marine | -0.410 | 0.285 | -1.44 | 0.150 |
| Environment: Terrestrial | -0.290 | 0.261 | -1.11 | 0.267 |
| Group: Fungi | -0.278 | 0.406 | -0.68 | 0.494 |
| Group: Invertebrates | -0.199 | 0.247 | -0.81 | 0.420 |
| Group: Microorganisms | -1.217 | 0.505 | -2.41 | 0.016 |
| Group: Plants | -0.015 | 0.236 | -0.06 | 0.949 |
| Group: Vertebrates | -0.138 | 0.245 | -0.56 | 0.572 |
| Experience: 5-10 y | 0.785 | 0.335 | 2.34 | 0.020 |
| Experience: <2 y | 1.421 | 0.537 | 2.65 | 0.008 |
| Experience: >10 y | 0.778 | 0.311 | 2.50 | 0.013 |
| Experience: No experience | 1.426 | 1.051 | 1.36 | 0.176 |
